# Remarkably High Dielectric Constant and Capacitance Density by Ni/ZrO_2_/TiN Using Nanosecond Laser and Surface Plasma Effect

**DOI:** 10.3390/nano15030246

**Published:** 2025-02-05

**Authors:** Wei Ting Fan, Pheiroijam Pooja, Albert Chin

**Affiliations:** Department of Electronics Engineering, National Yang Ming Chiao Tung University, Hsinchu 300, Taiwan

**Keywords:** High-κ, laser anneal, metal insulator metal, TiN, ZrO_2_

## Abstract

Rapid thermal annealing (RTA) has been widely used in semiconductor device processing. However, the rise time of RTA, limited to the millisecond (ms) range, is unsuitable for advanced nanometer-scale electronic devices. Using sub-energy bandgap (E_G_) 532 nm ultra-fast 15 nanosecond (ns) pulsed laser annealing, a record-high dielectric constant (high-κ) of 67.8 and a capacitance density of 75 fF/μm^2^ at −0.2 V were achieved in Ni/ZrO_2_/TiN capacitors. According to heat source and diffusion equations, the surface temperature of TiN can reach as high as 870 °C at a laser energy density of 16.2 J/cm^2^, effectively annealing the ZrO_2_ material. These record-breaking results are enabled by a novel annealing method—the surface plasma effect generated on the TiN metal. This is because the 2.3 eV (532 nm) pulsed laser energy is significantly lower than the 5.0–5.8 eV energy bandgap (E_G_) of ZrO_2_, making it unabsorbable by the ZrO_2_ dielectric. X-ray diffraction analysis reveals that the large κ value and capacitance density are attributed to the enhanced crystallinity of the cubic-phase ZrO_2_, which is improved through laser annealing. This advancement is critical for monolithic three-dimensional device integration in the backend of advanced integrated circuits.

## 1. Introduction

In the past decades, thermal annealing techniques have advanced toward shorter cycles and higher temperatures. Crystallization using a furnace requires high-temperature heat treatment, which results in long diffusion lengths and is unsuitable for small-channel electronic devices. Rapid Thermal Annealing (RTA) has been adopted to mitigate impurity diffusion as a replacement for furnace annealing. Modern spike RTA achieves a temperature ramp-up rate of 400 °C/s, effectively reducing thermal diffusion effects. However, one significant limitation of spike RTA is that the irradiation energy must exceed the energy bandgap (E_G_) of the materials. The UV-visible light irradiation energy used in RTA is insufficient for wide-bandgap dielectrics in the backend of Integrated Circuits (ICs). Although indirect heating via the Si substrate is possible, the annealing effect on backend devices remains inadequate. Furthermore, the maximum temperature for backend IC processes is restricted to 400 °C to prevent damage to frontend CMOS transistors.

Our group reported that a high-capacitance-density ZrO_2_ MIM capacitor can be formed by continuous wave (CW) Ar⁺ laser with a large thermal budget [1]. Besides, the mechanism of improvement using sub-energy bandgap annealing is unclear. Similar to pulsed RTA, the pulsed laser is the technology trend to further decrease the thermal budget rather than the CW case. This is especially important for backend devices fabricated above the frontend MOSFETs. The ultra-fast laser annealed processed ZrO_2_ MIM capacitors have higher capacitance density than high-κ SrTiO_3_ [2], HfO_2_/Al_2_O_3_ [3] and IC-implemented HfO_2_ [4] MIM devices. A comparative performance table of our work with the reported works is shown in Table 1 [2,3,4,5,6,7,8]. In ZrO_2_, intrinsic defects such as oxygen vacancies form a quasi-continuum of states in the band gap, which can trap electrons [9]. Also, by increasing the annealing temperature, the crystallinity of ZrO_2_ thin films is improved and influences the film surface roughness [10].

**Figure 1:**
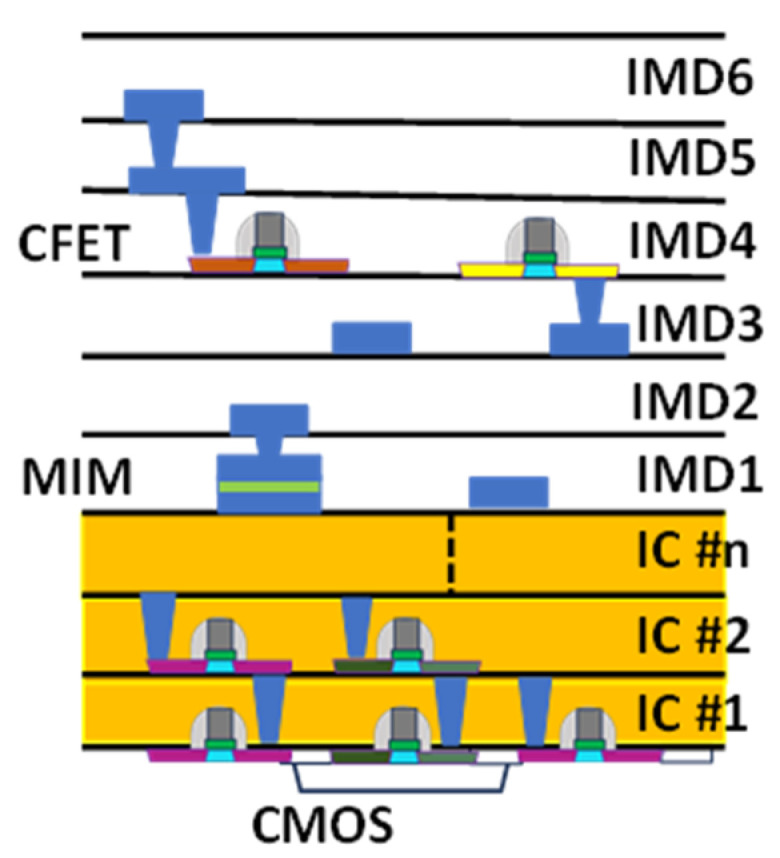
Monolithic Three-Dimensional (M3D) Integrated Circuit (IC) (IMD: Inter-Metal Dielectric.

In this study, we propose a novel annealing method to enhance device quality for backend devices on isolation oxide. A nanosecond (ns) pulsed laser is employed to control impurity diffusion further. The proposed approach was validated through a significant enhancement of 75 fF/μm^2^ capacitance density and a high dielectric constant (κ) of 67.8 using 15 ns laser annealing in the ZrO_2_ metal-insulator-metal (MIM) device. These represent the highest κ value and capacitance density reported in the literature [11,12,13,14,15]. It is noteworthy that the laser light energy is significantly lower than the energy bandgap (E_G_) of the material. The underlying mechanism involves the surface plasma effect [16], which generates high temperatures on the metal surface, effectively heating the ZrO_2_ dielectric. The temperature profile under various laser annealing conditions was calculated using Matrix Laboratory (MATLAB). The peak temperature at the TiN metal surface can reach up to 870 °C, efficiently annealing the ZrO_2_ dielectric. In contrast, the temperature drops sharply below 400 °C within a TiN thickness of 30 nm, ensuring minimal impact on frontend CMOS transistors. This technology offers a significant improvement in device performance for Monolithic Three-Dimensional (M3D) integration in the backend of advanced IC, as illustrated in Figure 1. The cross-section of interconnects, featuring ultra-thin body CMOS-on-insulator layers, is designed to mimic the structure of axons and dendrites in a biological brain. Such M3D integration is critical for enhancing circuit speed and reducing switching power consumption beyond the capabilities of the most advanced microprocessors [17,18].

## 2. Materials and Methods

A 50 nm TiN layer was first deposited and patterned as the bottom electrode for a metal-insulator-metal (MIM) capacitor using DC sputtering (British Microvac 450CB, Birmingham, Ion Tech. Ltd., London, UK). This was followed by NH_3_^+^ plasma treatment under various conditions: 2000 W for 60 s and 120 s, 2400 W for 60 s, and 2800 W for 60 s, respectively, to prevent capacitance-equivalent-thickness (CET) degradation by forming interfacial TiON during post-deposition annealing (PDA). Next, an 8 nm ZrO_2_ layer was deposited via atomic layer deposition (ALD) (Fiji-202 DCS, Cambridge NanoTech, Waltham, MA, USA), followed by O_2_ at 400 °C for 30 min to enhance its dielectric quality. ALD provides a self-assembly mechanism with atomic layer-by-layer growth and precise thickness control. A HIPPO mid-power Q-switched laser (532 nm) was applied at energy densities of 5.4 J/cm^2^, 16.2 J/cm^2^, and 21.6 J/cm^2^. Finally, a 50 nm Ni top electrode was deposited using e-beam evaporation (EBX-10C, ULVAC, Tokyo, Japan). The devices were characterized by capacitance density-voltage (C–V) measurements using an E4980A (Agilent, Santa Clara, CA, USA) and current density-voltage (J–V) measurements using an Agilent 4155B. The laser-annealed (LA) samples were analyzed using MATLAB (The MathWorks, Inc., Natick, MA, USA) to evaluate the temperature profile effectively. X-ray diffraction (XRD) (X’Pert Pro MRD, PANalytical, Almelo, The Netherlands), transmission electron microscopy (TEM) (JEM-2010F, JEOL, Tokyo, Japan), and atomic force microscopy (AFM) (D3100, Bruker, Billerica, MA, USA) were employed to assess the material quality and improvements.

## 3. Results

The heat diffusion equation governing the temperature evolution in both the TiN and ZrO_2_ layers is given by:

(1) $$\frac{\partial T}{\partial t}=\alpha \;\nabla^{2}T+\frac{Q}{\rho c_{p}}\;,$$

(2) $$\alpha =\frac{k}{\rho c_{p}},$$

(3) $$\nabla^{2}T=\frac{\partial^{2}T}{\partial r^{2}}+\frac{1}{r}\frac{\partial T}{\partial r}+\frac{\partial^{2}T}{\partial z^{2}}\;,$$

where *α* is the thermal diffusivity, ∇^2^*T* is the Laplacian of the temperature (describing the spatial derivatives of temperature in both radial and axial directions), *k* is thermal conductivity, *ρ* is material density, *c_p_* is specific heat capacity, and *Q* is the heat source term (which is zero for ZrO_2_, but non-zero in TiN due to laser absorption). The heat is in a Gaussian profile in the radial direction because the laser beam typically has a Gaussian intensity profile. In the radial direction, the heat source term is expressed as follows:

(4) $$Q=\frac{A_{TiN\;}T_{{ZrO}_{2}}E}{tA}\exp(-\frac{r^{2}}{2\sigma^{2}}),$$

(5) $$T_{{ZrO}_{2}}=\frac{{4n}_{1}n_{2}}{{{(n}_{1}{+n}_{2})}^{2}},$$

where *A_TiN_* is the absorbance of the TiN layer, *T_ZrO_*_2_ is the transmittance of ZrO_2_, *E* is laser energy, *A* is spot size, *t* is laser irradiation time, and *σ* is related to the focal radius, representing the width of the Gaussian beam profile, *n*_1_ is the refractive index of ZrO_2_, and *n*_2_ is the refractive index of air. The ZrO_2_ dielectric exhibited negligible absorption of laser energy, as its bandgap (5 to 5.8 eV) [19,20] significantly exceeds the photon energy of 2.33 eV. The temperature increase is attributed to the heat generated from the photon energy absorbed by the TiN layer.

**Figure 2:**
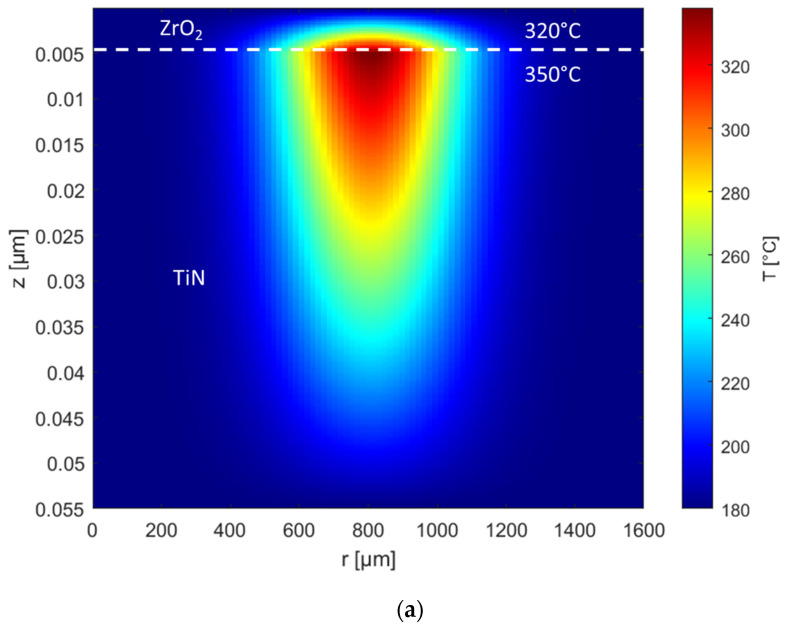

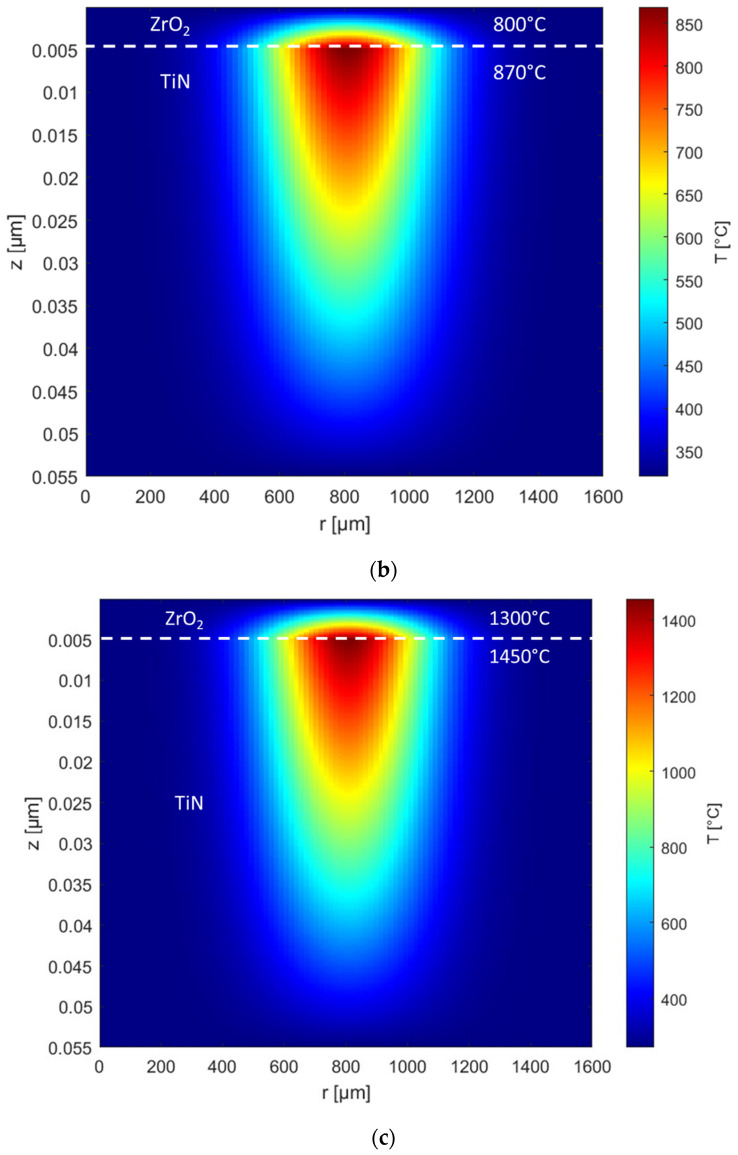
The temperature profile of TiN/ZrO_2_ samples under 532 nm laser of (**a**) 5.4 J/cm^2^, (**b**) 16.2 J/cm^2^, and (**c**) 21.6 J/cm^2^.

Figure 2a–c presents the simulation results of the temperature increase during laser irradiation with energy density fluences of 5.4, 16.2, and 21.6 J/cm^2^. TiN, a good plasmonic medium, is used as the metal layer to enhance the absorption of the irradiated pulsed laser and to diffuse heat to the ZrO_2_ insulator layer above it [21,22]. The calculations show that the average surface temperature of TiN increases to 350 °C, 870 °C, and 1450 °C, respectively, at these energy densities. As the heat diffuses, the corresponding temperatures at the ZrO_2_ layer are 320 °C, 800 °C, and 1300 °C. Furthermore, Lu et al. reported that ammonia (NH_3_) plasma pre-treatment is critical before depositing high-κ materials, as it significantly enhances interface properties [23]. Similarly, Edwards et al. observed that NH₃ plasma treatment prior to SiN deposition greatly improves the degradation characteristics of Al-GaN/GaN high-electron-mobility transistors (HEMTs) by reducing current collapse and eliminating gate lag after prolonged direct current bias [24]. This treatment strengthens bonds, making the structure more resistant to hot-electron damage and passivating defects caused by it.

**Figure 3:**
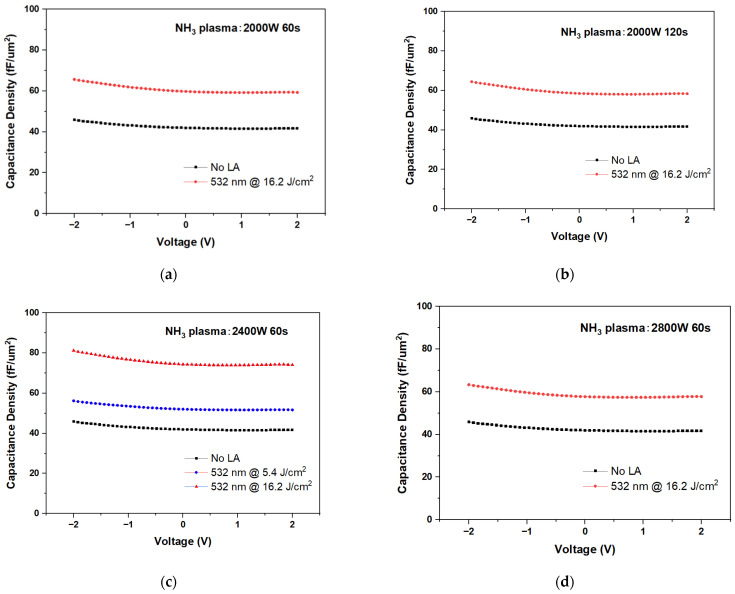
C–V of Ni/ZrO_2_/TiN MIM capacitors under NH₃⁺ plasma conditions (**a**) 2000 W 60 s, (**b**) 2000 W 120 s, (**c**) 2400 W 60 s, and (**d**) 2800 W 60 s (LA: Laser annealed).

Figure 3a–d shows the C–V characteristics of Ni/ZrO_2_/TiN MIM capacitors before and after laser annealing under NH_3_^+^ plasma treatment conditions at 2000 W (60 s and 120 s), 2400 W (60 s), and 2800 W (60 s) at 1 kHz. The use of a Ni electrode offers advantages, including a high work function and compatibility with reactive-ion-etching processes [25]. For 532 nm laser annealing combined with 2400 W, 60 s NH_3_^+^ plasma treatment (Figure 3c), the capacitance density increases monotonically with laser power, reaching 51.9 fF/μm^2^ for 5.4 J/cm^2^ and 75 fF/μm^2^ for 16.2 J/cm^2^. These values are significantly higher than the control devices before laser annealing, which exhibit a capacitance density of 41.7 fF/μm^2^ at −0.2 V. At low power (2000 W), NH_3_^+^ ions do not have enough energy to effectively incorporate nitrogen into TiN and higher power (2800 W) increases the kinetic energy of ions, which can introduce defects. Thus, the 2400 W, 60 s NH_3_^+^ plasma treatment enhances the formation and density of the nitridation layer, which effectively prevents the reaction between ZrO_2_ and TiN at high temperatures to form TiON. Under these conditions, the plasma treatment significantly improves the TiNx surface for laser annealing. Prolonged treatment times are also less effective compared to increased NH_3_^+^ plasma density. Thus, the 2400 W, 60 s NH_3_^+^ plasma condition represents an optimal balance for TiN surface nitridation, resulting in the best capacitance performance. Furthermore, when ZrO_2_/TiN is laser annealed at 532 nm with an energy density of 21.6 J/cm^2^, the temperature at the TiN surface rises to 1450 °C, and heat diffusion raises the ZrO_2_ temperature to 1300 °C. More than 10 devices of the same wafer were measured under the same conditions.

**Figure 4:**
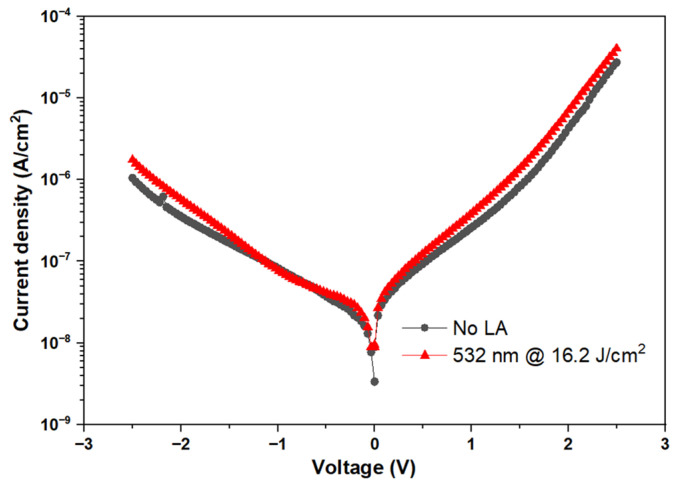
J–V characteristics of the Ni/ZrO_2_/TiN (LA: Laser annealed).

Figure 4 highlights the J–V plot of the Ni/ZrO_2_/TiN capacitor before and after laser annealing at 532 nm, 16.2 J/cm^2^. The leakage current increases slightly by 2.67 × 10^−8^ A/cm^2^ at −0.2 V than the control devices before laser annealing, with a leakage current of 2.17 × 10^−8^ A/cm^2^ at −0.2 V.

**Figure 5:**
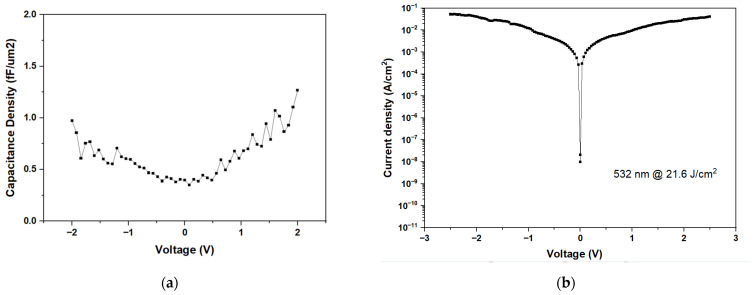
Ni/ZrO_2_/TiN MIM capacitors laser annealed at 532 nm, 21.6 J/cm^2^ (**a**) C-V and (**b**) J-V characteristics.

Figure 5a,b shows the C–V and J–V analyses, respectively, for Ni/ZrO_2_/TiN MIM capacitors under higher 21.6 J/cm^2^ laser annealing. No capacitance could be measured, and the Ni/ZrO_2_/TiN MIM device behaves like a small resistor. As shown in Figure 2c, the temperature at the TiN surface can rise to 1450 °C, and heat diffusion raises the ZrO_2_ temperature to 1300 °C. The laser energy is sufficiently high to induce thermal stress through local temperature rises that exceed the fracture strength of the film. This can lead to film wrinkling, cracking, or even detachment, resulting in device failure [26]. Although these temperatures remain below the melting points of ZrO_2_ and TiN, such high temperatures may cause reactions between ZrO_2_ and TiN, leading to bond breaking, the release of free Zr and Ti metals, and, ultimately, the shorting of the capacitor.

**Figure 6:**
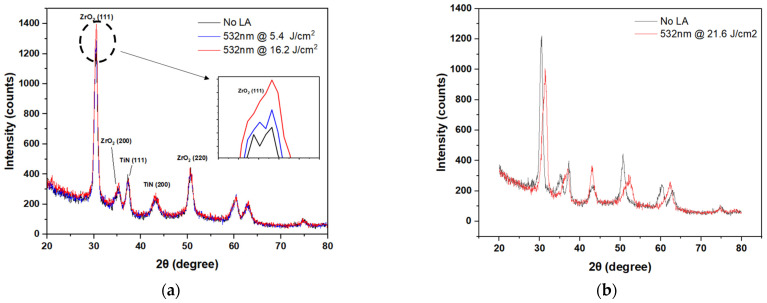
XRD patterns of the Ni/ZrO_2_/TiN capacitors without LA and LA at 532 nm (**a**) 5.4 J/cm^2^, 516.2 J/cm^2^ (inset maximized peak), and (**b**) 21.6 J/cm^2^ (LA: Laser annealed).

The slight increase in leakage current after laser annealing is attributed to larger grain size, as seen in XRD (Figure 6). The grain size of the ZrO_2_ can be calculated using the Scherrer formula as follows:

(6) $$D=\frac{k\lambda }{\beta \cos\theta }$$

where *D* is the grain size, *λ* is the X-ray wavelength, *k* = 0.9 is a dimensionless shape factor, *β* is the line broadening at half the maximum intensity (FWHM), and *θ* is the Bragg angle. From the Scherrer formula in Equation (6), the calculated grain sizes of ZrO_2_, before and after laser annealing at 532 nm, and 16.2 J/cm^2^ are 6.85 nm and 8.21 nm, respectively. These results further lead to higher capacitance density. This is similar to findings in TiO_2_ MIM capacitors [2]. Reduced crystalline peak with phase (111), shown in the inset of Figure 6a, results from laser annealing at 532 nm 5.4 J/cm^2^ and 21.6 J/cm^2^ (Figure 6b) due to either insufficient modification or excessive surface dislocation. The temperature increase with increasing laser energy density is due to the surface plasma effect, as shown in Figure 2. The higher temperature increases the crystallization of ZrO_2_ (Figure 6a), its κ value, and capacitance density. However, when ZrO_2_/TiN is laser annealed at 21.6 J/cm^2^, the TiN surface temperature surges to 1450 °C. At such a high temperature, thermal stress arises and leads to ZrO_2_ defects shown in Figure 6b and device failure.

**Figure 7:**
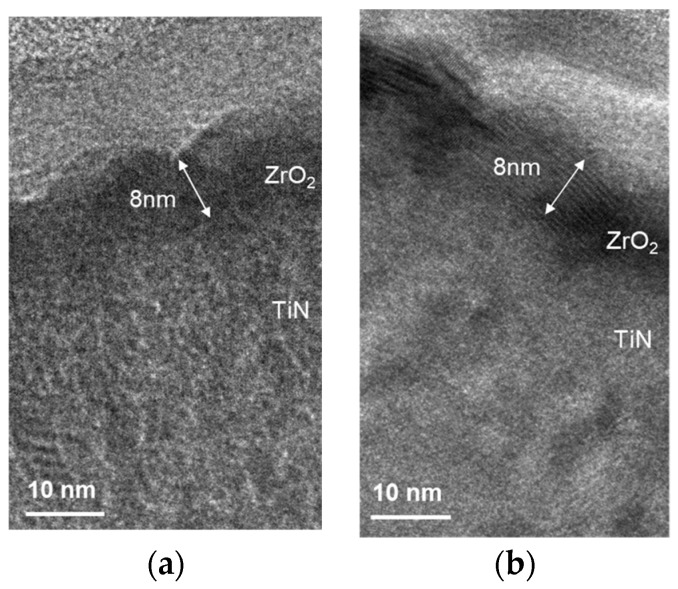
TEM cross-sectional image of the Ni/ZrO_2_/TiN capacitors (**a**) before and (**b**) after laser annealing.

The TEM analysis was used to study the laser annealing conditions. Figure 7a,b shows the cross-sectional TEM images of Al/ZrO_2_/TiN structures before and after laser annealing. Clearer crystallization of ZrO_2_ is observed after laser annealing, which is consistent with the XRD results. In the TEM image, the relatively rough top interface is made of Al rather than Ni. The samples analyzed by TEM should not contain magnetic substances such as iron, cobalt, nickel, or other similar materials.

**Figure 8:**
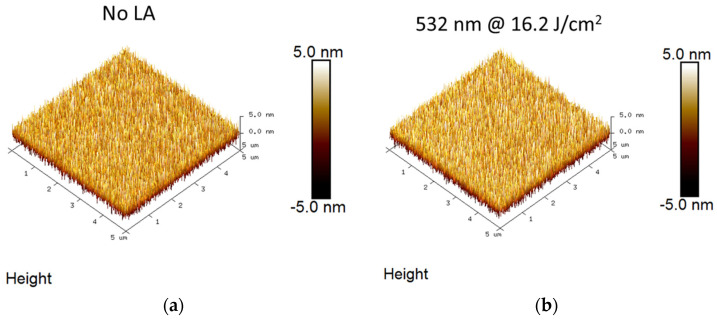
AFM images wide scans spectra of (**a**) No LA and (**b**) 532 nm LA, 16.2 J/cm^2^ (LA: Laser annealed).

The surface morphology of ZrO_2_ samples before and after laser annealing was studied using AFM images, as shown in Figure 8a,b, with 5 µm × 5 µm scans. The root-mean-square roughness of ZrO_2_ before laser annealing was found to be around 1.14 nm, whereas the roughness of ZrO_2_ after 532 nm laser annealing with 16.2 J/cm^2^ was 1.25 nm. During the laser annealing process, the rise in temperature enables grain boundary migration and promotes grain coalescence. More energy is available for atoms to diffuse, and lower surface energy grains enlarge at high temperatures. The significant grain growth observed in the XRD analysis highlights the enhanced surface roughness of the laser-annealed ZrO_2_ samples.

## 4. Conclusions

A high-performance Ni/ZrO_2_/TiN device has been achieved, offering both increased capacitance density and low leakage current. MATLAB calculations confirmed that the annealing temperature rises with increasing laser energy density, providing an alternative approach to achieving higher-κ dielectrics for next-generation MIM capacitors without the need to constantly introduce new materials.

## Figures and Tables

**Table 1:** Comparison of our work with reported works.

Dielectric	Dielectric Constant	Capacitance Density	Leakage Current Density	Voltage
ZrO_2_ (This work)	67.8	75 fF/μm^2^	4.06 × 10^−7^ A/cm^2^	1 V
SrTiO_3_ [2]	147	28 fF/μm^2^	3 × 10^−8^ A/cm^2^	2 V
HfO_2_/Al_2_O_3_ [3]	22	12.6 fF/μm^2^	10 nA/cm^2^	-
HfO_2_ [4]	-	43 fF/μm^2^	5 fA/μm^2^	1 V
HfO_2_/ZrO_2_/HfO_2_ [5]	43	7.4 µF/cm^2^	9.4 × 10^−5^ A/cm^2^	0.5 V
ZrO_2_/La_2_O_3_ [6]	33	30.5 fF/cm^2^	9.70 × 10^−7^ A/cm^2^	1 V
Ti-Doped ZrO_2_ [7]	-	12.21 fF/µm^2^	7.85 × 10^−7^ A/cm^2^	1 V
Al_2_O_3_/TiO_2_/Al_2_O_3_ [8]	-	18.3 fF/µm^2^	6.4 × 10^−9^ A/cm^2^	1 V

## Data Availability

The data presented in this study are available upon request from the corresponding author. The data are not publicly available due to privacy.
